# SG-APSIC1170: Reduction of hospital-onset MRSA bacteremia with chlorhexidine baths among MRSA-colonized patients

**DOI:** 10.1017/ash.2023.57

**Published:** 2023-03-16

**Authors:** Maria Theresa Cabahug, Theresa Cabahug, Li Jie, Foo Shi Yun, Wu Tuo Di, Chai Hairu, Harminder Kaur, Suhailah Binte Nasir

**Affiliations:** Changi General Hospital, Singapore; Singapore, Changi General Hospital, Singapore; Changi General Hospital, Singapore; Changi General Hospital, Singapore; Changi General Hospital, Singapore; Changi General Hospital, Singapore; Changi General Hospital, Singapore; Changi General Hospital, Singapore

## Abstract

**Objectives:** Methicillin-resistant *Staphylococcus aureus* (MRSA) is a major concern for hospitalized patients in Singapore. Hospital-onset (HO) MRSA bacteremia is monitored at the national level as an indicator of hospital quality. Patients who have colonized with methicillin-resistant *Staphylococcus aureus* (MRSA) are more likely to develop an MRSA infection in the future. A topical antiseptic solution or cloth called chlorhexidine gluconate (CHG) is effective against several gram-positive and gram-negative bacteria, including MRSA. **Methods:** The following control measures were present before and throughout the study period: (1) active screening of MRSA upon admission; (2) initiation of contact precaution once MRSA is detected; and (3) emphasis on strict hand hygiene. In January 2021, an intervention was for routine application of CHG bathing as follows: (1) training materials were developed; (2) train-the-trainer sessions were organized; (3) compliance regarding the application of CHG baths was monitored; and (4) the postimplementation process was reviewed. **Results:** There was no change of hand hygiene rate before and after implementation. In 2020, 17 cases of MRSA bacteremia occurred in the hospital, with an infection incidence of 0.54 per 10,000 patient days. In 2021, there were 10 cases of HO-MRSA bacteremia infection, with an overall rate of was 0.30 per 10,000 patient days. **Conclusions:** Daily bathing with chlorhexidine reduced the risk of MRSA acquisition and of hospital-acquired bacteremia.

